# Supplementary material for: Cell-Fate Determination from Embryo to Cancer Development: Genomic Mechanism Elucidated
Source: Int J Mol Sci. 2020 Jun 27;21(13):4581. doi: 10.3390/ijms21134581 (PMC7369777; doi:10.3390/ijms21134581)
Supplement: Supplementary file 1 [file ijms-21-04581-s001.zip › Supplementary Figure S1.docx]

**Supplementary Figure S1**

**Supplementary Figure S1 (HRG-stimulated MCF-7 cells):**

**A)** Fold change in ensemble (group) averages between expression groups shows that coherent-stochastic behavior (emergent coherent behavior from stochastic expression) represented by the center of mass (average) of group reveals a clear coil-globule transition.

**B)** Ensemble average of fold change in individual expressions between two temporal groups, <***c****^k^*(t_j+1_)/***c****^k^*(t_j_)>, does not reveal any transitional behavior that is attributable to the stochastic behavior of expression (sensitive in fold change).

**C)** Ensemble average of time difference in expression groups, <***c****^k^*(t_j+1_)- ***c****^k^*(t_j_)>, supports the coherent scenario in (**A**), where at around the CP (*ln*<*nrmsf*>~ -2.5) there is a positive maximum at 0-15 min and negative minimum at 0-20 min that corresponds to either an ON or OFF state, respectively.
